# Endocrine Disruption of Vasopressin Systems and Related Behaviors

**DOI:** 10.3389/fendo.2017.00134

**Published:** 2017-06-19

**Authors:** Heather B. Patisaul

**Affiliations:** ^1^Department of Biological Sciences, Center for Human Health and the Environment, NC State University, Raleigh, NC, United States

**Keywords:** bisphenol, oxytocin, sex differences, EDC, genistein, soy, social, anxiety, estrogens

## Abstract

Endocrine disrupting chemicals (EDCs) are chemicals that interfere with the organizational or activational effects of hormones. Although the vast majority of the EDC literature focuses on steroid hormone signaling related impacts, growing evidence from a myriad of species reveals that the nonapeptide hormones vasopressin (AVP) and oxytocin (OT) may also be EDC targets. EDCs shown to alter pathways and behaviors coordinated by AVP and/or OT include the plastics component bisphenol A (BPA), the soy phytoestrogen genistein (GEN), and various flame retardants. Many effects are sex specific and likely involve action at nuclear estrogen receptors. Effects include the elimination or reversal of well-characterized sexually dimorphic aspects of the AVP system, including innervation of the lateral septum and other brain regions critical for social and other non-reproductive behaviors. Disruption of magnocellular AVP function has also been reported in rats, suggesting possible effects on hemodynamics and cardiovascular function.

Endocrine disrupting chemicals (EDCs) have garnered considerable attention over the past few decades, partly because of their omnipresence, but also because of rapidly compounding evidence that exposure, particularly during critical windows of development, is likely contributing to increasing incidence of multiple chronic diseases. Because, historically, EDC research has focused on steroid hormone disruption, especially estrogen and androgen disruption, EDCs are most often thought of in the context of reproductive disorders including infertility, genital malformations, accelerated puberty, and reproductive cancer. But the concept of endocrine disruption is far more broadly inclusive of other hormones and their targets, including the neuropeptide hormones vasopressin (AVP) and oxytocin (OT).

Broadly, AVP and OT are found only in mammals, are structurally similar, and evolutionarily derived from the pituitary hormone, vasotocin (VT). Magnocellular AVP and OT are axonally released into the periphery from the paraventricular and supraoptic nuclei (PVN and SON) *via* the neurohypophysis and coordinate a range of physiological processes, including uterine contractions, milk letdown, blood pressure, thermoregulation, and osmotic balance. The parvocellular system sends projections to the median eminence and throughout the brain and is sexually dimorphic, steroid hormone sensitive, and fundamental for the coordination of affiliative and social behaviors including courtship, pair bonding, empathy, reciprocity, trust, and context-specific aggression (1–4). In addition, populations of AVP/OT-releasing neurons have been identified in other areas of the hypothalamus and in extrahypothalamic structures such as the medial amygdala (5). OT binds to the OT receptor (OTR), and AVP binds to one of two AVP receptor (AVPR) subtypes: AVPR1A or AVPR1B, the central distribution of which can differ substantially by sex, age, and species (5). Differences in the region-specific distribution of AVPRs and/or OTRs have been linked to individual and species variation in prosocial phenotypes including social attachment, parental behavior, and social anxiety, as have genetic polymorphisms in AVP, OT, and/or their receptors. The anatomical and functional features of AVP/OT neuronal subpopulations are beyond the scope of this review and detailed elsewhere (6, 7).

Centrally, the actions of OT and AVP largely overlap, albeit with some sexual dimorphisms, and these redundancies are not surprising given their relatively recent evolutionary divergence (summarized in Table 1). They can also have independent and even opposing effects. For example OT, but not AVP, appears to be critical for the extinction of social fear and promoting social interaction *via* enhancement of social preference suppression and social anxiety (8). Emerging evidence also suggests OT may suppress food intake and increase energy expenditure (9). Critically, opposing effects of AVP and OT refine the control of emotional behavior, and dysregulation of either can result in psychopathology. In general, central OT is anxiolytic, antidepressive, and prosocial, whereas AVP is anxiogenic and can heighten depressive-type behaviors (8). Consequently, there is concern that endocrine disruption of central AVP and/or OT function could adversely impact emotional control, and possibly heighten risk of psychosocial disorders. AVP and OT systems are also anatomically and functionally linked with catecholaminergic systems (10) and the mesolimbic dopamine system (1). That AVP and OT are fundamental to such a wide range of functions and behaviors indicates that disruption by EDCs could have profound and multifaceted effects throughout the neuroendocrine system. This review presents what is currently know about EDCs, AVP/OT systems, and the behaviors and physiological functions of these neuropeptides coordinate.

**Table 1:** Functions attributed to AVP/OT and thus possibly vulnerable to EDCs.

Oxytocin (OT)	Vasopressin (AVP)
**Neurohypophysial actions**	
↑ Parasympathetic autonomic functions	↑ Sympathetic and parasympathetic regulation
Milk letdown	↑ Vasoconstriction
Uterine contractions at parturition	↑ Blood pressure
**Central actions**	
↓ Aggression (♀ > ♂)	↑ Aggression and territorial behaviors
↓ Anxiety; ↑ relaxation, well-being, and trust	↑ Anxiety
↑ Initiation of social contact	↑ Attraction and partner selection
↑ Pair and social bonding	↑ Pair and social bonding (♂ > ♀)
↑ Partner preference formation (♀ > ♂)	↑ Partner preference (♂ > ♀)

## Evidence for Endocrine Disruption of Social Traits and Chemical Contributions to Psychosocial Social Disorders

Exploration and understanding of how EDCs may alter the organization and function of neuroendocrine systems outside of the hypothalamic–pituitary–gonadal (HPG) and thyroid axes is underdeveloped, particularly in mammals. This is perhaps surprising given the sensitivity of neuropeptide systems to steroid hormones, their sexually dimorphic properties, and their diverse functional roles in the brain and peripheral organs. Growing recognition that environmental factors are likely contributing to rapidly rising rates of psychosocial disorders in which neuropeptides are thought to play a central role, including autism spectrum disorders (ASDs), has ignited greater interest in understanding how EDCs might impact targets outside of the reproductive axis. For example, while disorders of the social brain clearly have a heritable component, there is growing consensus that genetics are not fully explanatory and may possibly only account for maximally half of risk. Genetic factors contribute only an estimated 30–40% of ASD heritability, with most of that attributable to common genetic variants (11). Thus, in the vast majority of instances, ASD and other disorders of the social brain undeniably result from a complex confluence of sex specific gene vulnerabilities layered with adverse, and critically timed, environmental interactions including chemical exposures. The challenge is figuring out which chemicals, and how a “perfect storm” of genetic predispositions and environmental insults manifests as clinical disease. Elucidating the specific mechanisms by which OT and AVP signaling pathways could be vulnerable to EDCs is considered critical to identifying and understanding possible linkages between chemical exposures and psychosocial disease risk.

There are likely upwards of 90,000 chemicals in our environment today, although a full accounting has proven to be nearly impossible, even for regulators such as the US Environmental Protection Agency (EPA) who are supposedly monitoring their potential toxicity, distribution, and use (http://cen.acs.org/articles/95/i9/chemicals-use-today.html). A subset of these is categorized as endocrine disrupting chemicals (EDCs) because of their potential to perturb endocrine systems. A consensus definition regarding what constitutes an EDC has proved elusive, and various definitions have been published each with similar but deliberately different wording (12). The precise language used for each is largely reflective of how the definition is applied and for what functional purpose it serves, particularly in a regulatory decision context. Because it was developed specifically for scientific purposes, this review will use the Endocrine Society definition, which states that an EDC is: “an exogenous chemical, or mixture of chemicals, that interferes with any aspect of hormone action” (13, 14). Thus, “exogenous substance” could be anthropogenic or naturally occurring [e.g., soy phytoestrogens (15)], and disruption of the organizational effects of hormones is considered most likely to result in permanent effects.

In their landmark 2006 review and call for action, environmental health scientists Philip Landrigan and Philippe Grandjean argued that industrial chemicals are undeniably contributing to the rapidly rising incidence of neurodevelopmental disorders, including ASD and attention deficit hyperactivity disorder (ADHD) (16). Although many of the chemicals identified as the most dangerous (including lead, methylmercury, and arsenic) have little to no endocrine disrupting activity, others are well known EDCs including the polychlorinated biphenols (PCBs) and many pesticides. In the subsequent decade, Landrigan and Grandjean, and numerous additional researchers have echoed and enhanced this call for greater investigation of the non-reproductive outcomes of EDC exposure, and emphasized the pressing need to understand how EDCs might contribute to impairments in reciprocal social engagements, repetitive/stereotypic behaviors, and other hallmark features of psychosocial and behavioral disorders (13, 17–21).

While it is not difficult to find broad speculation in the scientific and general literature that chemical exposures are contributing to rapidly rising rates of ASDs and ADHD, this literature is unfortunately peppered with falsely alarmist and unsubstantiated claims backed by weak or inconclusive data. Because many of these hyper-exaggerated linkages are frequently propagated by the media, there is confusion and even distrust regarding the risks EDCs pose to human health. *Direct evidence* linking any specific EDC to a clearly defined clinical disorder involving the social brain is sparse, and no single chemical has yet been definitively implicated (18, 21–25). For example, elevated prenatal androgens have been strongly associated with ASD risk for more than a decade (26–35) leading some to hypothesize that EDCs that alter androgen action may be contributory. That is clearly, however, and at the very least, only part of the story as supporting evidence in any experimental model system is extremely limited (22).

Thus, so little is known about the mechanisms by which the social brain is vulnerable to EDCs and other chemical exposures (20, 22, 36), particularly in humans, has proven to be a formidable obstacle when trying to convince regulators and other policy makers to enact actions which reduce EDC exposure. In addition, this mechanistic information gap is a significant barrier to efficiently and proactively screening chemicals for neurodevelopmental effects, or mitigating exposures that may be contributing to psychosocial disorders. Thus, gaining clearer understanding regarding the neural underpinnings of these possible linkages is of seminal importance.

Within the EDC field, and toxicology in general, compelling phenotypes drive subsequent mechanistic inquiry. Historically, the focus of EDC research has been on the HPG axis because the first and most profound exposure-related outcomes identified were reproductive. Effects included thinning eggshells in birds, altered sex ratios in turtle clutches, distorted courtship behavior in multiple avian species, abnormal gonadal and genital morphology in alligators, and numerous instances of intersex amphibians and fish (37–39). These and other worrisome and clearly adverse reproductive phenotypes drove a multidisciplinary quest to identify the endocrine disrupting mechanisms by which such outcomes arise. Invariably, disruption of steroid hormone action was found to be causal, particularly during critical developmental windows (39–41). Similarly, as the field has matured and expanded, heightened concern over rising rates of psychosocial disorders is now driving growing interest in the impact of EDCs on non-reproductive brain regions and the hormones that coordinate social behavior, including neuropeptides.

Although no animal model can fully capture the sophisticated complexity of human social behavior, the neuroendocrine pathways coordinating numerous social traits are highly conserved (7, 42), including the coordinating roles of AVP and OT. Behaviors such as play, maternal care, aggressive or competitive acts, reciprocal grooming, investigation of novel conspecifics, pair bonding, and social recognition are frequently modeled in animals to explore the neural underpinnings of human social behaviors and, by extension, how they might be susceptible to chemical exposures (39, 43–49). Because AVP/OT and the dopaminergic pathways they feed into are heavily influenced by sex steroids across the lifespan (50–59), it is highly plausible that their sexually dimorphic ontogeny and function may be particularly vulnerable to endocrine disruption. While sparse compared to available data on reproductive endpoints, evidence from a diverse range of species has revealed that targets critical to sociality and social cognition may indeed be vulnerable to environmental exposures, including the AVP/OT system (see Table 2 for a summary).

**Table 2:** EDCs shown to impact AVP/oxytocin (OT) pathways and related behaviors.

Chemical	Category/use	Effects	Primary mode of action	Reference
Bisphenol A (BPA)	Stabilizer in hard plastics and epoxy resins	Altered AVP and OT neuron numbers and innervation of sexually dimorphic regions associated with social and aggressive behaviors in multiple species; anxiogenic in multiple species	Estrogen disruptor	(60–66)
Chlorpyrifos	Insecticide	Altered hypothalamic AVP and OT levels (mRNA and protein); sexually dimorphic impacts on social, exploratory, and anxiety-related behaviors	Acetylcholinesterase inhibitor	(67, 68)
Dichlorodiphenyltrichloroethane (DDT)	Pesticide (restricted in the USA but still in use globally)	Demasculinized vasotocin innervation in Japanese quail	Estrogen and androgen disruptor	(69)
Genistein (GEN)	Isoflavone phytoestrogen found in soy and other legumes	Altered AVP and OT neuron numbers and innervation of sexually dimorphic regions associated with social and aggressive behaviors in multiple species; anxiogenic in males of multiple species	Estrogen and thyroid hormone disruptor	(69–74)
Methoxychlor	Insecticide	Abrogated male copulatory behavior in Japanese quail; disrupted female affiliative behavior in female prairie voles	Estrogen disruptor	(75)
Polybrominated diphenyl ethers (PBDEs)	Fire retardants (currently being phased out of use but rapidly replaced with structurally similarly compounds)	Impaired AVP release from the SON in response to dehydration; disruption of nitric oxide release related to AVP function in rats	Thyroid hormone disruptor	(76–78)
Polychlorinated biphenols (PCBs)	Now banned organochlorides used in many industrial applications including in paints, hydraulic fluids, lubricants, adhesives, pesticide mixtures, and sealants	Impaired AVP release from the SON in response to dehydration in rats	Estrogen and thyroid hormone disruptor	(76, 77)
Vinclozolin	Fungicide	Suppressed male copulatory behavior in Japanese quail	Androgen disruptor	(79)

## Early Evidence for Endocrine Disruption of AVP and OT Pathways

That the environment, including chemical exposures, impacts the neurohypophyseal nonapeptides was first and most comprehensively described in birds (79), particularly the Japanese quail (*Coturnix japonica*). Seminal research dating back decades has consistently and repeatedly shown that multiple aspects of the quail VT system is vulnerable to EDCs including bisphenol A (BPA), diethylstilbestrol (DES), and high doses of the phytoestrogen genistein (GEN) (69, 70). In mammals and birds, the number of AVP (VT in birds) neurons and projections in the bed nucleus of the stria terminalis (BnST) and amygdala are markedly greater in males. This is one of the most consistently observed neural sex differences across taxa, and these projections are well known to coordinate sexually dimorphic social and reproductive behaviors (80). A series of studies led by Giancarlo Panzica has elegantly demonstrated the profound sensitivity of this system to embryonic manipulation by estrogens or estrogenic EDCs. Exogenous administration of estrogen, diethylstilbestrol (DES), or an aromatase inhibitor during incubation fully blocked male copulatory behavior at puberty and induced the complete sex reversal of VT-ir in the preoptic area, BnST, and lateral septum (LS) (70, 81). Reduced male copulatory behavior was also observed following embryonic exposure to high doses of the phytoestrogen genistein (GEN) (69), which is one of the most potent EDCs on nuclear estrogen receptors (ERs), particularly ERβ (82, 83). Demasculinization of VT-ir was induced by embryonic GEN, and *p*,*p*′-DDE [a long-lived metabolite of dichlorodiphenyltrichloroethane (DDT)] (69). Other EDCs also shown to suppress male copulatory behavior following embryonic exposure, purportedly *via* interference with estrogen and androgen pathways, include atrazine (herbicide), methoxychlor (pesticide), and vinclozolin (fungicide) (79).

Another relatively early example of endocrine disruption in the OT/AVP system used a vole model (*Microtus*) (75). Within this genus, some species are spontaneously more prosocial (prairie and pine voles; *Microtus ochrogaster* and *Microtus pinetorum*, respectively) than their promiscuous relatives (montane and meadow voles; *Microtus montanus* and *Microtus pennsylvanicus*, respectively), and rats or mice. Social attachment is rare in mammals but a hallmark of human social interactions. Foundational research in voles has uncovered the significance of neuropeptidergic regulation of social behaviors including paternal care, pair bonding, and maternal aggression (3, 45). In the socially monogamous pine vole, perinatal oral exposure to approximately 2,000 µg/kg bw methoxychlor produced some offspring effects, but only in females (75). A non-significant trend toward increased time spent alone in the partner preference test and less aggression toward a strange male was interpreted to indicate a reduced preference for the mate and a disruption in affiliative behavior. OTR binding was unchanged in LS but reduced in the cingulate of the exposed females compared to unexposed controls. This region is thought to play a key role in stress responses and emotional processing (84). In their conclusions, the authors advocated for wider use of the vole model because “monogamous mammals share a common reproductive pattern of long-term bond. The pine vole may prove to be a new and important model for species displaying monogamy, including humans (75).” Surprisingly, no one in the EDC community heeded this call, and no work was subsequently performed in the vole model until we began our own line of investigation with BPA and prairie voles nearly a decade after this pioneering study.

Overall, the literature on EDCs and AVP/OT pathways remains small, and work has primarily focused on a small subset of high priority chemicals, most of which are known disruptors of estrogen signaling pathways. Yet, it highlights the importance of exploring alternative modes of action when thinking about neurotoxicants and EDCs. Three representative examples are discussed in depth below.

## Bisphenol A (BPA)

There is perhaps a no more notorious or thoroughly studied EDC than BPA. Present in polycarbonate plastics, the epoxy lining of canned foods, thermal paper, and other common household products, BPA is classified as a “high volume production” compound and continuous low level (resulting in mean blood levels of 4 ng/ml or lower) exposure is virtually ubiquitous and unavoidable (85). Although frequently characterized as “weakly estrogenic” BPA effects are multi-modal. For example, we have repeatedly shown that developmental exposure to doses as low as 2.5 µg/kg bw sex specifically alters the mRNA expression of ERα and ERβ in sexually dimorphic hypothalamic and limbic subnuclei in the neonatal and older rat brain (86–90). Neurobehavioral outcomes purportedly mediated *via* androgen receptors and epigenetic effects have also been reported (91–93). The dose defined as the “no adverse effect level” for systemic toxicity and thus the dose level below which biologically meaningful effects purportedly do not occur is 5 mg/kg bw per day. The level considered “safe” for human exposure is extrapolated from this level and is 50 µg/kg bw per day according to the US EPA, and 4 µg/kg bw per day in the European Union.

Information regarding BPA-related impacts on nonapeptide pathways is limited, but we and others have generated some evidence, in a diverse range of taxa, showing that developmental exposure to BPA alters the organization and function of AVP and OT pathways. In a study done in collaboration with Andrea Gore’s laboratory, we found that Wistar rats perinatally exposed to BPA *via* drinking water [1 mg/L; a dosing regimen that resulted in serum levels approximately equivalent to humans (94)], displayed elevated anxiety-related behaviors as juveniles (60). Consumption of a soy-rich diet, which is hormonally active and contains estrogenic phytoestrogens including GEN, ameliorated the behavioral effects to some degree. This outcome was somewhat surprising. We had predicted that BPA and soy would have additive effects because of their similar modes of action. Interactions with diet may explain some of the inconsistencies in the BPA literature regarding effects on brain and behavior (93, 95). Nevertheless, linkages between developmental BPA exposure and an anxiogenic phenotype have repeatedly been shown in dozens of studies using a variety of animal models and human populations (representative examples include (62, 91, 93, 96–100)). Acceptance of this outcome by the risk assessment community has been tentative because the causal mechanism(s) remains unclear (94). In our Wistar rat study, exposure decreased ERβ and Mc4r expression levels in the amygdala of both sexes. These genes play crucial roles in regulating the production and release of AVP and OT in the PVN. Specifically, agonism of Mc4R in magnocellular neurons induces dendritic secretion of OT (101), an effect that is anxiolytic (102, 103). As a follow-up to our initial study, expression levels were subsequently examined in the PVN of the same Wistar rats using identical methodology. There was some evidence for downregulation of AVP mRNA in BPA-exposed females, but the effect did not quite reach statistical significance (*p* ≤ 0.07), and no effect on OT, AVP1aR, OTR, or Mc4R expression was observed in either sex (*unpublished observations*). As was observed in the amygdala, BPA produced no effects on any genes of interest in rats reared on the soy-rich diet, highlighting the significance of other environmental factors, including diet, when seeking evidence of endocrine disruption. Lack of effects on transcription does not necessarily indicate lack of effects on translation, transport, or release, and it is possible that only subpopulations of PVN neurons are susceptible, necessitating an experimental approach with great anatomical resolution to assess possible effects. In addition, AVP and OT are released *via* multiple mechanisms (2, 5), sometimes simultaneously, including volume diffusion through the extracellular space following release from large dense core vesicles, widespread circulation through the ventricular system, and targeted release into specialized extrahypothalamic regions, any or all of which might be vulnerable to EDCs.

In a separate study, we showed that that neonatal exposure to BPA (50 mg/kg bw or 50 µg/kg bw) by subcutaneous injection can alter the number of PVN OT immunolabeled cells in adulthood (in female rats) (61). Internal BPA levels were not assessed for this study, but because injection bypasses first metabolism, circulating BPA levels were undoubtedly higher than the study described above and higher than typical human levels. Exposure significantly increased OT-immunoreactive (-ir) neuron numbers, but only in the anterior PVN, a result interpreted to potentially indicate sequestration of OT and reduced release from nerve terminals. Similar outcomes were observed in a subsequent study using the prairie vole model (62). Animals were orally exposed over PNDs 18-14 to 5, 50, or 50,000 µg/kg bw and tested as juveniles or adults. Females in the highest exposure group had fewer OT-ir neurons in the posterior PVN but more AVP-ir neurons in the anterior PVN. At the lower two doses, BPA eliminated the well-characterized sex difference in PVN TH-ir neuron numbers and reversed it at the highest dose. This effect was mirrored by similar alterations in social investigation. In this species, males are typically more inclined to interact with a novel animal than females. At the two lower doses, BPA eliminated this sex difference and at the highest dose, reversed it. Disruption of pBnST TH-ir neuron numbers also occurred at the lowest dose, but not the higher two making that outcome somewhat more difficult to interpret. These data are not in complete directional accord with our rat study, an outcome that could result from the different exposure window, doses used, or species differences in OT/AVP pathways. Ongoing studies are underway to try and resolve these differences.

A series of studies led by Emilie Rissman using C57BL/6J mice suggests that BPA-related effects on OT and AVP signaling pathways may be multi- and transgenerational. In this model, the dams (F0) are exposed to BPA during pregnancy so offspring (F1) exposure is gestational. The subsequent generation (F2) is exposed as developing germ cells in the ovaries of their embryonic F1 parents. Thus, this generation is also “directly” exposed. The F3 generation is the first generation regarded as unexposed and thus the first set of offspring in which truly transgenerational effects can be assessed (63). Mice reared on a diet delivering approximately 170 µg/kg bw BPA (to the dams) during gestation displayed social deficits, particularly in females, that coincided with a decrease in AVP mRNA expression levels in whole embryonic brains obtained from their ED18.5 siblings (both sexes). AVP levels were also decreased in the F4 embryos of both sexes albeit to a lesser degree (64, 65). Reduced OT mRNA expression was observed in the F4 males. Evidence of effects on social behavior, including juvenile social recognition and social investigation, was reported but directionally inconsistent across generations with the F1 generation showing heightened social investigation and the F3 generation displaying reduced responses to novel females, and the F4 mice more actively engaged with their social peers than those from an unexposed lineage (104). A subset of adult F1 and F3 mice were examined *via* immunohistochemistry to asses if BPA had altered sexually dimorphic AVP-ir levels in several brain regions. F1 males had fewer AVP-ir in the MePD than unexposed males, but females were unaffected. No evidence of disrupted AVP-ir levels was found for the MePD, PVN, LS or BnST (66).

Collectively, these findings support the hypothesis that BPA exposure may disrupt the organization of AVP/OT pathways arising in the PVN, thereby impacting related social behaviors. They also suggest, unsurprisingly, that specific outcomes likely differ between sex and species depending on the degree to which they are prosocial. Additional environmental factors, including diet, may modify outcomes and contribute to discordance across studies. Experiments with a greater emphasis on linking cause and effect are greatly needed to estimate the degree to which BPA may affect AVP/OT systems in humans.

## Chlorpyrifos (CPF)

Organophosphate pesticides make up approximately 70% of all pesticides in the USA and are developmental neurotoxicants related to nerve agents such as Sarin and VX. Although their primary mechanism of action is inhibition of acetylcholinesterase, they can also have endocrine disrupting properties, particularly at dose levels which are more environmentally relevant and well below those which cause systemic toxicity (16, 22, 105). CPF has been linked to ADHD and other developmental neural disorders in children, prompting the EPA to impose a ban on residential use in 2001 (22, 106, 107). A complete ban was recommended in 2016 but has not been implemented. CPF will thus remain in use for golf courses, and about 50 different types of crops, including corn, soybeans, row crops (such as broccoli), and fruit trees. Work in mice and rats has repeatedly shown that developmental exposure results in long-lasting (and sometimes sex specific) effects on emotional functions, activity, learning, serotonergic and dopaminergic transmission, neuronal differentiation, and synaptogenesis (108). Collectively, these effects implicate peptidergic pathways as a possible target for endocrine disruption.

Data in support of CPF as a peptidergic disruptor are sparse but suggestive of sex-specific vulnerability. In CD1 mice, pre- and/or postnatal exposure to CPF resulted in a dose-dependent decrease in hypothalamic AVP at 5 months of age concomitantly with increased OT (67). This outcome was more pronounced in males than females and following pre-, rather than post-, natal exposure. The same group subsequently reported effects on OT and AVP mRNA expression in the amygdala as well as the hypothalamus (68). Notably, ERβ was also found to be upregulated in the male hypothalamus. In both studies CPF-exposed mice displayed altered social and exploratory behaviors, with the specific outcomes differing by sex. Collectively, these data are consistent with a robust body of evidence showing that CPF is a developmental neurotoxin and endocrine disruptor, and a likely environmental contributor to ASD, ADHD, and other behavioral disorders. Additional work in other species is needed to provide resolution regarding the degree to which CPF can interfere with AVP and OT signaling pathways.

## Phytoestrogens

Not all EDCs are anthropogenic. Soy and other legumes contain isoflavone phytoestrogens, which are used to aid in the recruitment of nitrogen-fixing bacteria but are also well-characterized xenoestrogens (83, 109, 110). GEN, for example, is found in soy-based and soy-supplemented foods, including soy infant formula, and can be purchased as a dietary supplement. Evidence of the endocrine disrupting properties of GEN dates back decades, and soy is so well recognized as a hormonally active food, that it is frequently advertised and promoted as such (15, 111–115). As with BPA and other manufactured EDCs, the majority of GEN studies have focused on reproductive endpoints (although with the distinctive difference that, historically, most studies presume possible outcomes will be “beneficial” because soy is “natural” while BPA is not), but there are some data showing endocrine disruption of AVP-related pathways and systems, including behaviors (112).

Although initially thought to be anxiolytic (116), long-term consumption of soy-rich diets has been shown to enhance aggression in male cynomolgus monkeys (117) and Syrian hamsters (72), the latter of which also had lower AVP1A expression in the LS but higher AVP1A expression in lateral hypothalamus. My lab has also shown that two dietary isoflavone supplements produced anxiolytic elevated plus maze behavior in proestrus female rats, but anxiogenic responses in gonadally intact males (118). Similarly, male rats maintained on a diet containing 150 µg/g GEN and daidzein displayed increased anxiety and elevated stress-induced plasma AVP and corticosterone levels (71). Elevated hypothalamic AVP content (measured by ELISA) has also been reported in rats maintained on a diet containing 1,250 ppm GEN (73). Sexually dimorphic AVP-ir in the rodent brain can also be altered by early-life exposure to GEN, including at doses akin to the levels found in soy infant formula. For example, in CD1 mice, oral intake of 50 µg/kg GEN in the first week of life slightly, but significantly increased AVP-ir in the female BnST but did not impact the sexually dimorphic AVP innervation of the LS (74). Notably, the sexually dimorphic density of AVP-ir neurons in the medial parvicellular part of the PVN (PaMP) was eliminated by postnatal GEN, with higher numbers in females and lower numbers in males.

Dietary consumption of soy is globally increasing, meriting greater understanding of its endocrine disrupting properties, particularly in infants and young children. Although sex-specific isoflavone-related effects on the rodent AVP/OT system have been sporadically shown by multiple laboratories, including my own, work in this area remains limited and incomplete. In some regards, this is because work on “natural” EDCs is notoriously difficult to obtain funding for. There is also a problem of perception. While concerns about BPA, fire retardants, and other manufactured EDCs remains high, phytoestrogens are some of the most potent xenoestrogens humans regularly are exposed to. Arguably, they are one of the most significant but underappreciated EDCs of concern.

## Endocrine Disruption of AVP/OT Pathways: A Role for ERs

How chemicals interact with and perturb AVP/OT pathways are undoubtedly multi-modal, but action on ERs, and disruption of ER expression, is likely a primary mechanism by which BPA, GEN, and other EDCs influence AVP/OT pathways. That non-classical EDCs like CPF can have disruptive effects on AVP/OT action, hint at alternative modes of action but most work to date has focused on estrogen-disrupting compounds because the organization and function of AVP/OT signaling pathways are exquisitely sensitive to steroid hormones. We have repeatedly shown that developmental BPA exposure can perturb ERα and ERβ gene expression throughout the rat hypothalamus and components of the mesolimbic dopamine system, including the PVN and BnST (86–90), across the lifespan. Moreover, in the transgenerational mouse model, one of the most striking findings was disrupted ERα-ir in the F3 females from the BPA-exposed lineage, with higher levels in the AVPV and lower levels in the BnST (66). The phytoestrogen coumestrol (found in clover and other pasture legumes) and GEN upregulate ERβ mRNA expression in the PVN, an effect opposite to that of 17β-estradiol (119, 120). If and how these neural effects translate to adverse behavioral phenotypes remains to be established.

How estrogens and androgens contribute to the sexual differentiation and function of the AVP/OT system are sex and species dependent and associated with functional differences in prosociality (121, 122). Manipulation of OT/AVP levels *via* direct exposures to exogenous hormones, agonists, or alteration of the social environment can significantly modify the number of OT, AVP, and TH neurons in the PVN, thereby resulting in anxiety-like behavior and alterations of prototypical male and female sociosexual behavior (123–127). Similarly, neonatal manipulation of estradiol or testosterone alters prairie vole affiliative behaviors later in life, and estradiol administration during adulthood alters estrus and locomotor activity (57, 128, 129). Males gonadectomized on the day of birth, for example, fail to form a pair bond after AVP administration (52).

Pathways coordinating social recognition are well known to be regulated by estrogen, with both ERα and ERβ knockout mice showing social impairments (130, 131), and ERβ knockout females failing to generate OT or AVP mRNA expression in response to exogenous estrogen administration (132, 133). Although the functional role of limbic ERβ remains ambiguous, ERβ in the PVN and associated structures, including the BnST, plays a fundamental role in mediating motivational and anxiety-related behaviors (134–136). ERβ has also been identified as one component of a “four-gene micronet” regulating social recognition (7, 131) *via* the PVN, amygdala, and olfactory system. By this model, estradiol simultaneously acts through ERβ in the PVN to increase OT, and ERα in the amygdala to increase OTR expression. This view is consistent with the observation that the density of ERα markedly differs across prosocial and asocial species and is directly related to the degree to which individuals and species are prosocial (3, 137, 138). Conceptualizing and testing endocrine disruption of ER-sensitive AVP pathways as a system, such as this micronet, while simultaneously accounting for species and sex differences, would further holistic understanding of how EDCs affect non-reproductive behaviors.

Finally, a subpopulation of parvocellular PVN OT and AVP neurons, along with ERβ and coordinating input from the BnST, is thought to be involved in the stress response (135, 139, 140) supporting the possibility that EDCs may influence the hypothalamic–pituitary–adrenal axis. Surprisingly, this is a neglected area of EDC research. Given the comparatively higher promiscuity of ERβ (relative to ERα) for ligands including multiple phytoestrogens including GEN (82), and even the androgen metabolite 5α-androstane-3β,17β-diol (3β-diol) (141), this is a strikingly understudied but likely avenue for endocrine disruption.

## AVP Impacts—Beyond Brain and Behavior

In both rats and prairie voles, we have shown that BPA alters only specific subpopulations of OT and AVP neurons. Although we surmised that the populations of OT-ir and AVP-ir neurons impacted by BPA are primarily parvocellular, the possibility that BPA alters the density and function of magnocellular neurons could not be ruled out. This would suggest an avenue for homeostatic disruptions including cardiovascular effects and hypertension associated with BPA and, by extension, other EDCs (142, 143). Intriguingly, in humans, BPA exposure has been tentatively linked with cardiovascular disease and hypertension, but the causal mechanisms remain to be characterized (144).

Long before its role in social behavior was identified, AVP was termed the “antidiuretic hormone.” Magnocellular AVP neurons in the PVN and SON release AVP both centrally and systemically in response to dehydration, hemorrhage, and stress and are known to be one of the primary physiological regulators of water-electrolyte balance (145). These AVP neurons receive osmosensitive inputs from the organum vasculosum lamina terminalis *via* glutamatergic synapses and are modulated by nitric oxide (NO) signaling pathways (146–148). Work using adult male rats and SON tissue punches has yielded evidence that the PCB mixture Aroclor 1254 and the structurally related polybrominated flame retardants [polybrominated diphenyl ethers (PBDEs)], including the pentabrominated mixture DE-71, significantly reduced AVP release from the SON in response to dehydration (76, 77). Subsequent work revealed that perinatal oral exposure to 1.7 or 30.6 mg/kg/day resulted in elevated systolic blood pressure responses at 3 h post-hyperosmotic challenge, an effect the authors interpreted as possibly attributable to decreased plasma AVP levels (78). A single paper also suggests that long-term exposure to chlorobenzenes (solvents) may also disrupt neurohypophysial AVP and OT release (149). Complementary work by multiple research teams has identified NO signaling as a potent target of many EDCs including PBDEs, PCBs, and other organohalogens (representative examples include (74, 150, 151)) suggesting another possible route by which EDCs could impact AVP function. Concomitant work focused on the parvocellular aspects of AVP signaling has also found evidence of NO endocrine disruption by GEN, BPA, and other EDCs (39, 74, 151).

## Summary and Conclusion

Although available data are sparse there is compounding evidence that EDCs can alter the ontogeny and function of AVP and OT signaling pathways critical to social behavior and, possibly, osmotic balance and other peripheral functions. Rising rates of behavioral, hypertensive, and other disorders for which AVP are known to play a coordinating role suggest a causal role for environmental factors, including chemical exposures, but which specific ones remains elusive. The complex chemical landscape we all face and inevitably invades us makes it challenging to identify the individual or subset of compounds that pose the greatest health risks to this and subsequent generations. Data from a myriad of species from birds to mammals, however, suggest that EDCs that interfere with estrogen signaling (including BPA, GEN, PBDEs, and PCBs) are plausible disruptors of AVP/OT pathways. Compared to what is known about how these EDCs alter the sex specific organization and function of reproductive neuroendocrine pathways, investigation of nonapeptide disruption is in its relative infancy, but a topic of heightening interest and exploration. Moreover, only a handful of compounds have been tested for AVP-related outcomes at all.

Going forward, there is a compelling need to understand and develop effective screening methods for EDC activity, particularly on non-steroid hormone targets, to get a better handle on what possible consequences this chemical class may pose to human health. As this paper went to press the EPA’s endocrine disruptor screening program (EDSP), an effort that took more than two decades to construct, and only recently reached the capacity to effectively identify any chemical as a purported EDC, was slated for elimination. If cut, this would leave absolutely no mechanism for screening any extant or pre-market chemicals for any EDC activity of any kind. The assays within the EDSP are reasonably effective at targeting sex steroid hormone disruptors but ineffective at identifying non-steroidal endocrine disrupting activity including OT and AVP disruption (152) leaving a desperate need for improvement. Within the USA there is absolutely no federal-level mechanism in place to evaluate where EDCs are found, let alone regulate their use in common items such as cosmetics, personal care products, food containers or durable goods. Consequently, daily exposure is virtually silent and unceasingly increasing (12). Moreover, there is an ever-churning conveyer belt of worrying chemical replacements such that when EDCs, such as the PBDEs, DDT, and BPA, are finally phased out, they are rapidly replaced by structurally similar compounds. In this regard, there is a compelling need for ongoing inquiry regarding EDC activity, particularly on non-traditional targets such as AVP systems.

Future work focusing on richly estrogen sensitive limbic populations known to confer individual, sex and species differences in prosocial traits, especially the BnST, is critically needed to better understand how AVP/OT signaling pathways and, consequently, social behaviors might be vulnerable to endocrine disruption. Much EDC work, particularly on social and other non-reproductive behaviors, remains largely descriptive with limited understanding of causal relationships. Elucidating specific mechanisms of endocrine disruption is essential for establishing causality between exposure and adverse behavioral outcomes. Maximizing the probability of success will hinge on the selection of the most appropriate animal model for the specific question being addressed, and the translational value of the outcome for human neurophysiology. Neuroendocrinologists familiar with a wide range of taxa are well poised for critical discovery because classical toxicology still overly relies on dated testing strategies and traditional rat models. Incredibly, toxicology is still struggling to incorporate transgenic animals and other more modern approaches now considered basic tools of neuroscience (153, 154), leaving vast opportunity for the neuroendocrine community to make critical discoveries toward the goal of uncovering the chemical contributions to social disorders and other debilitating conditions such as cardiovascular disease and hypertension.

## Author Contributions

The sole author completed this manuscript independently.

## Conflict of Interest Statement

The author declares that the research was conducted in the absence of any commercial or financial relationships that could be construed as a potential conflict of interest.
